# Chronic Inflammation and Glycemic Control: Exploring the Bidirectional Link Between Periodontitis and Diabetes

**DOI:** 10.3390/dj13030100

**Published:** 2025-02-26

**Authors:** Jyotsna Suresh Ranbhise, Songhyun Ju, Manish Kumar Singh, Sunhee Han, Salima Akter, Joohun Ha, Wonchae Choe, Sung Soo Kim, Insug Kang

**Affiliations:** 1 Department of Biochemistry and Molecular Biology, School of Medicine, Kyung Hee University, Seoul 02447, Republic of Korea; jogm25@khu.ac.kr (J.S.R.); thdgus8543@khu.ac.kr (S.J.); manishbiochem@gmail.com (M.K.S.); sunheehan@khu.ac.kr (S.H.); aktersalima@gmail.com (S.A.); hajh@khu.ac.kr (J.H.); wchoe@khu.ac.kr (W.C.); 2 Biomedical Science Institute, Kyung Hee University, Seoul 02447, Republic of Korea; 3 Department of Biomedical Science, Graduate School, Kyung Hee University, Seoul 02447, Republic of Korea

**Keywords:** severe periodontitis, diabetes mellitus, microbial dysbiosis, inflammation, hyperglycemia, innate immunity

## Abstract

Periodontitis and diabetes mellitus are two highly prevalent chronic conditions that share a bidirectional relationship, significantly impacting public health. Periodontitis, a gum inflammation caused by microbial dysbiosis, aggravates glycemic control in diabetics, while uncontrolled diabetes heightens periodontitis severity. These conditions create a vicious cycle, where inflammation and microbial dysbiosis mutually drive disease progression, exacerbating systemic health. The underlying mechanisms involve inflammation, immune dysfunction, and microbial dysbiosis, with both diseases contributing to a chain of chronic inflammation that exacerbates systemic health. This relationship is significant because managing one condition can significantly impact the other. In diabetic individuals, interventions such as periodontal therapy have shown effectiveness in improving glycemic control, underscoring the potential of integrated strategies for managing these conditions simultaneously. In this review, we highlight the importance of a deeper understanding of the molecular and immunological interactions between these diseases is essential for developing integrated therapeutic approaches, with the potential to enhance the quality of life of the patient significantly.

## 1. Introduction

Periodontitis and diabetes mellitus are two prevalent chronic conditions with significant public health impacts. Both are interconnected through a bidirectional relationship that affects overall health, posing a growing clinical challenge. Periodontitis, the most common disease of the oral cavity only after dental caries [1], is a multifactorial, chronic inflammatory disease that leads to the progressive destruction of periodontal tissues, including the gingiva, periodontal ligament, and alveolar bone [2]. It is associated with many systemic diseases and conditions, but the association of periodontitis with diabetes mellitus (DM) is so common that it is known as the sixth complication of DM [3]. Diabetes Mellitus is a group of metabolic diseases marked by persistent high blood sugar levels brought on by impairments in either the action or secretion of insulin or both over a prolonged period [4]. It is usually associated with overweight and obesity, aging, ethnicity, and familial history [5]. The global prevalence of both these diseases is alarming. According to the Global Burden of Disease Study (2021), around 1 billion people were affected by severe periodontitis [6,7,8], across different regions in the world with a global age-standardized prevalence of 12.50% [6]. Whereas, the prevalence of diabetes mellitus was 10.5% affecting over 536.6 million people majority of which account for type 2 DM [9]. The World Health Organization (WHO) predicts that diabetes will become the seventh leading cause of death by 2030 [10], primarily due to its association with cardiovascular diseases, stroke, and kidney failure. Studies have shown that periodontitis and diabetes mellitus influence each other. Periodontitis can worsen glycemic control in diabetic patients, while uncontrolled diabetes contributes to the progression of periodontal disease [11]. Chronic periodontitis-mediated insulin resistance and worsening of glycemic control are due to systemic inflammation and the release of pro-inflammatory cytokines such as TNF-α and IL-6 and other mediators by impairing insulin signaling pathways [12]. The growing evidence of the interaction between periodontitis and diabetes has direct clinical implications, impacting the management of both conditions and requiring integrated therapeutic approaches to improve patient health [13]. On the other hand, poorly controlled diabetes, particularly Type 2 Diabetes(T2D), can promote periodontal disease progression by impairing immune responses, reducing the efficiency of neutrophil function, and altering the composition of the oral microbiome, leading to dysbiosis [14]. Research indicates that advanced glycation end-products (AGEs) and their receptors (RAGEs) play a critical role in chronic inflammation and impaired wound healing, making diabetic patients more susceptible to tissue damage [11]. These interaction mechanisms have significant implications for the simultaneous treatment of these conditions, requiring a multidisciplinary approach that considers systemic inflammation, insulin resistance, and oral microbiome modulation. Thus, this review aims to describe the mechanism of pathophysiology linking these two conditions and to highlight the clinical consequences of the diagnosis and treatment of both.

## 2. Periodontitis

Periodontitis is an inflammatory disease of the supporting structure of teeth which includes gingiva, periodontal ligament, and alveolar bone, caused due to microbiota and results in alveolar bone and tooth loss [15]. Inflammation of the gingiva is termed as gingivitis and it precedes periodontitis in many cases [16]. According to the new consensus of the AAP/EFP, gingivitis is considered when the total percentage of bleeding on probing (BoP) is >10%. Periodontitis is considered to be manifested when interproximal CAL (clinical attachment loss) was detected in two or more interproximal sites not adjacent or there was an interproximal CAL of 3 mm or more, non-vestibular or lingual/palatal, for ≥2 teeth [17]. Additionally, diabetes, respiratory infections, cardiovascular disease, and other systemic disorders are all significantly exacerbated by periodontitis [14]. Pathogenic biofilms, or plaque, build up on the surface of teeth. These biofilms contain microbial dysbiosis populations that promote periodontitis [18]. Microbial dysbiosis, an imbalance in the microbial community, plays a critical role in the development of periodontitis [19]. Over time, the persistent bacterial challenge triggers a host immune response that, if dysregulated, results in tissue destruction [20]. Although microbial dysbiosis is a key etiological factor of periodontitis, its mere presence is insufficient for the disease initiation. When microbial dysbiosis, along with other risk factors such as genetics, environmental influences, and smoking combine, the full manifestation of the disease occurs [21]. The key microbial drivers of periodontitis include *Porphyromonas gingivalis*, *Treponema denticola*, and *Tannerella forsythia* [22]. These pathogens induce an inflammatory cascade through the activation of Toll-like receptors (TLRs). This cascade leads to the upregulation of pro-inflammatory cytokines and matrix metalloproteinases (MMPs), which contribute to tissue degradation and alveolar bone resorption [23]. To understand periodontitis at a molecular level, it is essential to examine the microbial and immune mechanisms that drive the disease.

### 2.1. Pathophysiology of Periodontitis

#### 2.1.1. Microbial Dysbiosis

The healthy human oral microbiome is home to an estimated more than 700 kinds of bacterial species [24]. Although the oral microbiome is predominantly composed of bacteria the presence of fungi, viruses, and archaea is a common site, pointing towards the complex network of microbial synergic or antagonist interactions among them [25]. Bacteria are the most abundant and diverse, with members of certain phyla, such as *Firmicutes*, *Bacteroidetes*, *Actinobacteria*, *Fusobacteria*, and *Proteobacteria*, being particularly prevalent [26]. Age, genetics, nutrition, hygiene, and general health influence the oral microbiome’s composition, with some species being more prevalent in particular people [27]. Despite the large number of species, a relatively small subset of these bacteria (around 20–30 species) is typically responsible for most oral diseases like dental caries and periodontitis [28] when the microbiome balance is shifted to the state of dysbiosis. This small subset was categorically placed into 5 different bacterial complexes first in 1998 by Socransky. Among these *Porphyromonas gingivalis*, *Treponema denticola* and *Tannerella forsythia* are the most well-characterized pathogenic species, often called the Socransky’s red complex [29]. These bacteria are strongly associated with active periodontal disease and have clear virulence factors that enable tissue destruction, immune evasion, and inflammation [30]. However, an increasing body of studies surrounding periodontal pathogens recognized that periodontitis is not solely attributed to the red complex bacteria [29]. Other microorganisms, such as *Aggregatibacter actinomycetemcomitans* and *Fusobacterium nucleatum*, an obligate anaerobic gram-negative bacillus, also play significant roles in disease progression [31]. *Aggregatibacter actinomycetemcomitans*, for instance, is implicated in aggressive periodontitis due to its ability to invade host tissues and induce immune dysregulation [32]. *Fusobacterium nucleatum*, while not part of the red complex, acts as a key bridging organism, facilitating the coaggregation of other pathogenic bacteria, thus contributing to the establishment of a pathogenic biofilm [33]. These species, along with other potentially pathogenic microorganisms, interact within the microbial community to exacerbate periodontal inflammation. Notably Various studies have named *Porphyromonas gingivalis* the main culprit in periodontal disease. Gram-negative, non-motile *P. gingivalis* is an asaccharolytic bacterium [34] that expresses a variety of virulence factors, such as lipopolysaccharides (LPS), extracellular trypsin-like proteases (gingipain), and fimbriae, that promote *P. gingivalis* to aggregate with different microbial communities, promoting the formation of dental biofilms [35]. The asaccharolytic feature of *P. gingivalis* refers to its ability to break down proteins and ferment amino acids to generate cellular energy [36]. *P. gingivalis* gingipains produce pilus appendages called fimbriae, anchored to the bacteria’s outer membrane [37]. Long fimbriae are formed from FimA protein subunits and achieve invasion by binding themselves to human glyceraldehyde 3-phosphate dehydrogenase (GAPDH), triggering host response by releasing pro-inflammatory cytokines. The second type, short fimbriae, is constructed from mfa1 structures and binds to other bacteria’s SspA and SspB proteins in oral biofilm. Major fimbriae FimA is believed to have a substantial role in this microbe’s colonization of the oral cavity. As virulence factors, they attack essential extracellular matrix components, impairing the epithelium’s barrier function and enabling *P. gingivalis* to access subepithelial tissues through the destruction of periodontal tissue and the breakdown of iron-binding proteins [37,38]. They divide the protein genetic sequences from lysine or arginine residues: lysine-gingipain, arginine-gingipain A, and arginine-gingipain B [39]. *P gingivalis* the main pathogen that causes the majority of disease progression is a late colonizer of the complex process of bacterial aggregation, and it works in synergy with early colonizers like *Streptococcus oralis, Streptococcus mitis*, *Streptococcus gordonii*, *Streptococcus*, *Streptococcus sanguis* and intermediate colonizer such as *Fusobacterium nucleatum* which guarantees, directly or through Treponema denticola, the adhesion of *P. gingivalis* [40]. While microbial dysbiosis is a key factor in periodontitis, the immune response exacerbates tissue destruction. Pathogenic microorganisms trigger a cascade of pro-inflammatory cytokines, leading to the activation of matrix metalloproteinases (MMPs) and osteoclasts, which drive bone resorption [41].

#### 2.1.2. Host Immune Response

The host immune response exhibited in periodontitis is predominantly inflammatory [42]. When the immune system detects microbial pathogens, it activates innate and adaptive immune responses [43]. In the early stages of infection in the presence of periodontal pathogens, the innate immune system acts via epithelial cells, phagocytes (neutrophils, macrophages, dendritic cells), the complement system, pattern recognition receptors (PRRs), and the production of pro-inflammatory cytokines and chemokines. Each component contributes to the body’s ability to recognize and respond to the microbial challenge.

##### Epithelial Cells and Barrier Function

**Figure 1:**
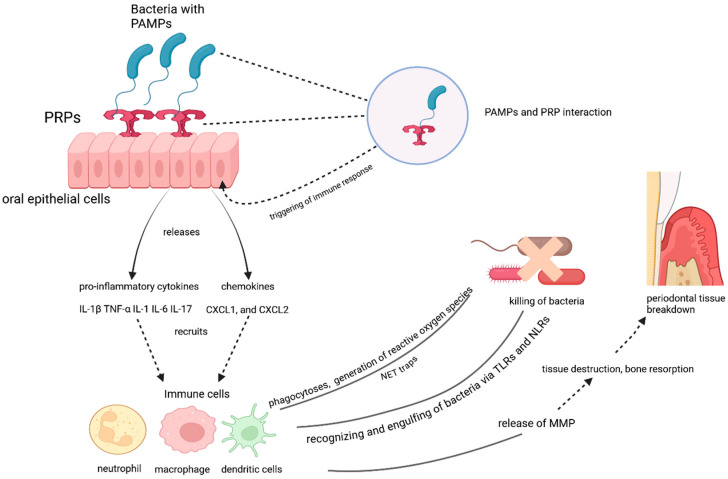
The role of oral epithelial cells and immune responses in periodontal tissue breakdown.

The oral epithelium serves as the first physical barrier against microbial invasion. In the presence of periodontal pathogens, epithelial cells lining the gingiva and oral mucosa are essential in initiating the immune response [44]. Epithelial cells contain pattern recognition receptors (PRRs), which recognize pathogen-associated molecular patterns (PAMPs) unique to microorganisms. Common PAMPs include bacterial components like lipopolysaccharide (LPS) from Gram-negative bacteria (e.g., *P. gingivalis*), peptidoglycan, and flagellin [45]. When PAMPs interact with PRRs (Toll-like receptors TLRs), the epithelial cells recognize the presence of pathogens and trigger immune responses (Figure 1). Upon detecting microbial components, epithelial cells release pro-inflammatory cytokines and chemokines to draw immune cells to the site of infection [46]. Interleukin-8 (IL-8) is a potent chemo stimulator for neutrophils, the first responders in periodontal disease [47]. Interleukin-1β (IL-1β) and Tumor necrosis factor-α (TNF-α) contribute to the amplification of inflammation and tissue destruction by promoting the production of matrix metalloproteinases (MMPs) and osteoclast activation [48]. CXCL1 and CXCL2 are the chemokines that further enhance the recruitment of neutrophils and other immune cells [49]. When the barrier function of the epithelium is compromised due to persistent bacterial infection, the permeability of the gingival tissues increases, allowing bacterial infiltration into deeper tissues and further amplifying the inflammatory response [50]. Pathogens like *P. gingivalis* can invade epithelial cells directly, further modulating the host immune response to their advantage and helping them persist in the environment [51].

##### Phagocytes in Periodontitis: Neutrophils and Macrophages

Phagocytic cells, which include neutrophils, macrophages, and dendritic cells, play a critical role in defending the host against the microbial threat in periodontitis. Neutrophils are the first responders to microbial invasion in the gingiva and play a crucial role in controlling the bacterial load during the early stages of periodontitis [52]. Numerous studies have also demonstrated the importance of neutrophils in preserving the periodontal tissue’s internal environment. [53]. They are attracted to the site of infection by cytokines and chemokines (e.g., IL-8) released by epithelial cells and resident immune cells. In phagocytosis, neutrophils recognize bacterial pathogens through their PRRs (including TLRs) and phagocytose them into intracellular vesicles [54]. This process is followed by killing bacteria by generating reactive oxygen species (ROS) and releasing antimicrobial peptides like defensins and cathelicidins [55]. Neutrophils also release neutrophil extracellular traps (NETs), which consist of DNA, histones, and antimicrobial proteins. NETs trap and neutralize bacteria, but excessive NET formation can also contribute to tissue damage and inflammation [56]. In addition to combating pathogens, neutrophils release Matrix Metalloproteinases (MMP)s, which degrade the extracellular matrix and contribute to the breakdown of periodontal tissue [57]. The continuous activation of neutrophils in periodontitis results in collateral tissue damage.

Macrophages are another critical phagocytic cell type in the immune response to periodontal pathogens. Derived from monocytes that migrate from the bloodstream into the inflamed periodontal tissues. Macrophages can perform several functions, including pathogen clearance, cytokine production, and tissue repair [58]. Like neutrophils, macrophages recognize and engulf bacteria through PRRs such as TLRs and NOD-like receptors (NLRs). Macrophages are key producers of cytokines, including IL-1β, TNF-α, and IL-6, which sustain the inflammatory response and recruit additional immune cells [59]. Macrophages also produce chemokines such as CCL2 (MCP-1) to attract monocytes and other immune cells to the site of infection. Macrophages also release MMPs, which contribute to the remodeling of the extracellular matrix [60]. These enzymes and cytokines exacerbate tissue destruction in periodontitis, including bone resorption.

##### The Complement System

The complement system is a key component of the innate immune response and is involved in the recognition and clearance of pathogens and the amplification of inflammation [61]. Proteolytic cleavage and successive activation of several proteins make up the complement system, which starts a chain of reactions [62]. That results in the lysis of pathogens, recruitment of immune cells, and enhancement of phagocytosis. In periodontitis, antibodies bind to bacterial antigens (antibody-antigen complexes) when the classic pathway is triggered, activating the complement component—C1q subunit of C1 [62]. When The Lectin Pathway is triggered, the proteins bind to restricted carbohydrate structures or acetylated compounds on the surface of pathogens [63], such as those found in *P. gingivalis*. The Alternative Pathway is a spontaneous activation of complement components, which is stabilized by microbial surfaces, leading to the deposition of complement proteins on the bacterial cell surface. In periodontitis, the complement pathway serves by opsonization mainly via C3b complement protein, binding to pathogens and marking them for phagocytosis by neutrophils and macrophages [63]. Chemotaxis, C5a, a potent anaphylatoxin, enhances the recruitment of immune cells to the site of infection, promoting inflammation and exacerbating tissue damage. Furthermore, the lysis of Pathogens by the formation of the membrane attack complex (MAC), through the activation of C5b-C9, directly lysing bacterial cells, although this process is not as prominent in periodontitis due to the protective nature of the oral biofilm.

The interaction between pathogen-associated molecular patterns (PAMPs) and pattern recognition receptors (PRRs) on oral epithelial cells triggers an immune response. The activation of PRRs leads to the release of pro-inflammatory cytokines (e.g., IL-1β, TNF-α, IL-6, IL-17) and chemokines (e.g., CXCL1, CXCL2), which recruit immune cells such as neutrophils, macrophages, and dendritic cells. These immune cells recognize and engulf bacteria via Toll-like receptors (TLRs) and NOD-like receptors (NLRs), generating reactive oxygen species and forming neutrophil extracellular traps (NETs) to kill bacteria. However, the immune response also releases matrix metalloproteinases (MMPs), contributing to tissue destruction, bone resorption, and periodontal tissue breakdown.

##### Inflammatory Mediators: Cytokines and Chemokines

In periodontitis, pro-inflammatory cytokines and chemokines play crucial roles in the recruitment and activation of immune cells. Cytokines IL-1β is one of the first cytokines immune cells produce upon pathogen detection. IL-1β promotes inflammation and activates the production of MMPs, leading to tissue destruction [64]. Next, TNF-α a potent cytokine that activates other immune cells and induces the production of inflammatory mediators. TNF-α plays a key role in bone resorption and is implicated in periodontal attachment loss [43]. IL-6 cytokine is involved in acute-phase response and contributes to systemic inflammation. It also promotes the differentiation of osteoclasts, leading to bone loss in periodontitis [65]. When the innate immune system cannot stop microbial invasion T cells and B cells are activated to target specific pathogens in the adaptive immune response, which is more extended and highly specific than the innate immune response, which is non-specific and instantaneous [66]. In periodontitis, the adaptive immune system plays a critical role in amplifying the immune response and attempting to control the chronic infection. However, in the context of periodontitis, this response can become dysregulated and contribute to tissue destruction rather than the resolution of the disease.

T cells are central to the adaptive immune response in periodontitis. Specifically, Th1 and Th17 subsets are involved in the inflammatory response [67]. Th1 cells release IFN-γ, which activates macrophages and enhances inflammation. Th17 cells secrete IL-17, which promotes the recruitment of neutrophils and further amplifies the inflammatory response. However, excessive Th17 activation is associated with tissue damage and bone resorption [68]. The cytotoxic T cells (CTLs) may play a role in eliminating infected tissue; their overactivation can also contribute to tissue damage from chronic inflammation [69]. B cells produce antibodies against specific periodontal pathogens. In periodontitis, antibodies (such as IgG) are produced in response to bacterial antigens, helping to neutralize pathogens and facilitate their clearance [70]. However, in periodontitis, antibodies alone do not always lead to pathogen clearance. The antibody response can be insufficient or may even contribute to tissue destruction by promoting the activation of inflammatory mediators like complement and matrix metalloproteinases (MMPs). T cells and B cells produce a variety of cytokines that regulate the immune response. In periodontitis, producing pro-inflammatory cytokines such as TNF-α, IL-1, IL-6, and IL-17 contributes to chronic inflammation, tissue degradation, and bone resorption [71]. The cytokines and inflammatory mediators now interfere with insulin signaling by inhibiting the insulin receptor’s activity or downstream signaling pathways. This reduces glucose uptake in cells, leading to insulin resistance—a key feature of type 2 diabetes. Furthermore, reactive oxygen species (ROS) generated due to the immune response also further disrupt insulin signaling and damage tissues, leading to the manifestation of Diabetes Mellitus.

#### 2.1.3. Oxidative Stress

Microbial dysbiosis and host immune response contribute to the major pathogenesis of periodontitis but the increasing body of studies surrounding oxidative stress cannot be ignored. Hence, we have to take into consideration the hyperactivated polymorphonuclear neutrophil phenotype that appears to be associated with periodontal disease [72]. Inflammatory cytokines cause the release of ROS from hyperactive PMN via the NADPH oxidase pathway [73]. These abundant Ros further activate other defense cells and osteoclast production leading to tissue destruction and bone resorption [74].

## 3. Diabetes Mellitus

The two primary forms are Type 1 diabetes (T1D) and Type 2 diabetes (T2D). Type 1 diabetes, often referred to as autoimmune diabetes, is a chronic illness caused by insulin insufficiency as a result of pancreatic β-cell loss, which causes hyperglycemia [75]. Conversely, Type 2 diabetes is the most common form of diabetes in adults [76]. It is primarily a result of insulin resistance, where the body’s tissues fail to respond to insulin effectively, leading to impaired glucose homeostasis [77]. The pathophysiology involves a complex interaction between genetic susceptibility and environmental triggers [78]. T2D accounts for approximately 90–95% of all diabetes cases, while T1D represents a smaller proportion. Globally, the disease burden is disproportionately high. Diabetes, particularly type 2 diabetes (T2D), is characterized by the deficiency of insulin caused by pancreatic β-cell dysfunction and insulin resistance in target organs [79].

### 3.1. Pathophysiology of Diabetes

#### 3.1.1. Insulin Resistance

Insulin is a peptide hormone secreted by the β-cells of the pancreatic islets of Langerhans that facilitates glucose uptake into cells, thereby regulating normal blood sugar levels in the body [80]. However, in diabetes, this process is disrupted, leading to elevated blood glucose levels, which have widespread effects on various organs and tissues, including the oral cavity. Insulin resistance is a key pathophysiological feature of type 2 diabetes [81] that occurs when the body’s cells become less sensitive to the effects of insulin and its downstream metabolic actions under normal serum glucose concentration [82].

Insulin resistance has been mainly related to high-fat and high-carbohydrate diets leading to fat mass gain in overweight and hence often related to obesity, particularly visceral fat accumulation, which leads to the release of pro-inflammatory cytokines like TNF-α, IL-6, and resistin [83]. These cytokines interfere with insulin signaling and promote chronic low-grade inflammation [84]. The increased demand for insulin overwhelms the pancreatic beta cells, leading to a relative insulin deficiency. This results in higher blood glucose levels (hyperglycemia), a hallmark of diabetes.

Interestingly in some literature, the competition to bind to glycoprotein receptor INS-R between insulin and TNF-α is also noted and cited as a cause of insulin resistance. Upon isolation of the insulin-binding fraction of rat liver membrane in 1970 it was clear that insulin acted through cell surface receptors [85]. There are more than 70 potential serine/threonine phosphorylation sites known in the Insulin receptor substrate (IRS) protein [86]. These receptors are not solely used by insulin and can bind to other molecules. In a highly inflammatory condition of periodontitis elevated TNF-α a pro-inflammatory cytokine, binds to IRS and impairs insulin signaling by serine phosphorylation of IRS-1 [87,88] and reduces GLUT-4 expression [89,90]. In periodontitis, TNF-α outnumbers the insulin molecule binding to the INS-R, thereby inhibiting insulin’s ability to activate the receptor and initiating glucose uptake, resulting in a state of insulin resistance.

#### 3.1.2. Inflammatory Response

Chronic hyperglycemia in diabetes has numerous detrimental effects on the body. High blood glucose levels contribute to the formation of advanced glycation end-products (AGEs), which result from non-enzymatic reactions between glucose and proteins or lipids [91]. AGEs accumulate in tissues and contribute to the development of diabetic complications by binding to receptors for advanced glycation end-products (RAGEs), triggering a pro-inflammatory response [91]. This leads to the activation of various inflammatory pathways, including the nuclear factor kappa B (NF-κB) pathway, which further promotes the release of pro-inflammatory cytokines. The persistent state of low-grade inflammation in diabetes contributes to tissue damage, including damage to blood vessels, nerves, and other organs. This chronic inflammatory state also influences the immune response, impairing the ability to resolve infections and leading to poor wound healing [92].

#### 3.1.3. Effects of Hyperglycemia

Hyperglycemia has been shown to impair immune function in several ways. High glucose levels inhibit neutrophil function, essential for clearing bacterial infections. In addition, diabetes alters the function of macrophages and T cells, which are critical for mounting an appropriate immune response [93]. These immune dysfunctions contribute to an increased susceptibility to infections, including periodontitis. Additionally, the pathophysiology of diabetes has a direct impact on oral health, establishing a bidirectional relationship between diabetes and periodontal diseases.

## 4. The Bidirectional Relationship

Diabetes, particularly type 2 diabetes, has a profound impact on oral health, and the relationship between diabetes and periodontitis is long recognized in the literature. Diabetes directly does not cause infectious oral conditions but the amalgamation of immunity inhibition and poor glycemic control can stimulate the development of periodontal disease in the host [94].

**Figure 2:**
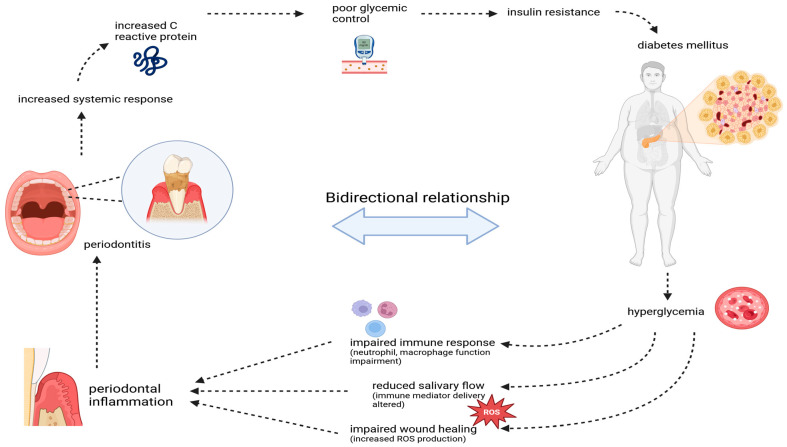
The bidirectional relationship between periodontitis and diabetes mellitus. Periodontitis-induced inflammation elevates systemic markers like C-reactive protein, worsening glycemic control and insulin resistance, contributing to diabetes progression. In turn, hyperglycemia in diabetes impairs immune function, reduces wound healing, and increases oxidative stress, exacerbating periodontal inflammation and tissue damage, creating a vicious cycle.

Hyperglycemia in diabetes impairs the immune function by causing neutrophil dysfunction, which makes individuals with diabetes more susceptible to infections, including periodontal disease In a hyperglycemic environment, neutrophil chemotaxis (the process by which neutrophils are attracted to the site of infection) and phagocytosis (the ability of and other immune molecules [95]. Wound healing is a complex and regulated neutrophils to engulf and kill pathogens) are especially impaired [96], which reduces the ability to control bacterial growth in the periodontal tissues. Hyperglycemia also affects macrophage function, leading to an increased production of inflammatory cytokines [97]. Hyperglycemia also reduces the salivary flow rate and impairs the delivery of immune mediators to the oral cavity, making it harder for the body to control infections. Moreover, the quality of saliva in diabetic patients can also be altered, with a reduction in the antimicrobial peptides process that involves inflammation, tissue remodeling, and repair [98]. In diabetes, chronic hyperglycemia increases the production of ROS (free radicals), which damages cellular structures such as proteins, lipids, and DNA [99]. This oxidative stress interferes with the normal wound-healing process by hindering fibroblast proliferation, collagen synthesis, and extracellular matrix formation. Delayed wound healing seen in diabetic patients also increases the risk of secondary infections, including gums and periodontal ligament infections, exacerbating periodontal disease progression and making it more challenging to manage (Figure 2). Furthermore, chemokines such as IL-8, MCP-1, and CXCL-1 are also elevated in the periodontal tissues of individuals with diabetes [100]. The persistent hyperglycemia in DM leads to the formation of covalent adducts between glucose, proteins, DNA, and lipids via the Maillard reaction leading to the formation of irreversible advanced glycated end-products AGEs [101]. In diabetic patients, the incremented production of AGEs accumulates in the tissues leading to structural and functional alterations [102]. In periodontal disease, AGEs bind to the extracellular matrix (ECM) of the basement membranes of the gingival tissue [103]. Accumulated AGE-modified collagen brings microvascular changes (thickens the basement membrane) which impairs the membrane’s permeability, hindering the transport of oxygen, metabolic products, phagocytes, and antibodies, to the affected tissue [2,104,105]. As a result, the tissues in the periodontal region become more vulnerable to injury, exacerbating inflammation and eventually contributing to the progression of periodontal disease. AGEs also bind to receptors for advanced glycation end-products (RAGEs) on immune cells, such as macrophages, neutrophils, and endothelial cells. This interaction triggers the production of pro-inflammatory cytokines and reactive oxygen species (ROS), further aggravating inflammation in the periodontal tissues [102]. Furthermore, AGEs also contribute to the cross-linking of collagen fibers in the periodontal tissues, making them stiffer and more resistant to normal tissue remodeling [106,107]. This impairs the healing process and promotes the breakdown of the periodontal attachment apparatus, leading to a more significant loss of connective tissue and bone. AGEs have been shown to enhance the activation of osteoclasts, the cells responsible for bone resorption [108]. In periodontitis, this leads to increased alveolar bone loss, contributing to the clinical severity of the disease in diabetic individuals.

In the case of Periodontitis, the inflammatory mediators released as part of this immune response can have significant systemic effects, exacerbating conditions like diabetes. The primary systemic impact of periodontal infections is an increased inflammatory burden on the body, which can negatively influence metabolic functions [109]. The chronic inflammation caused by periodontitis has several direct and indirect mechanisms that impair glycemic control in diabetic patients [110]. These mechanisms primarily involve insulin resistance, which makes it harder for the body to manage blood glucose levels effectively. Insulin resistance is key to elevated blood glucose levels. Periodontal disease-induced inflammation amplifies insulin resistance by increasing the levels of inflammatory cytokines [111]. These cytokines interfere with insulin signaling pathways, disrupting normal insulin receptor function and impairing glucose uptake into cells. Chronic inflammation from periodontitis has been shown to induce activation of the NF-κB pathway, leading to an increase in pro-inflammatory cytokines that directly impair insulin action [112]. This creates a vicious cycle, where poor glycemic control exacerbates periodontal disease, and periodontal disease, in turn, worsens glycemic control. Oxidative stress, which is elevated in periodontitis, can also impair insulin signaling by causing damage to the insulin receptor and other key components involved in glucose metabolism [113] (Figure 2).

### 4.1. Studies Shows Improvement in Glycemic Control After Periodontal Treatment

Clinical studies have demonstrated that treating periodontal disease can effectively improve glycemic control in individuals with type 2 diabetes [114]. Periodontal treatment helps reduce the systemic inflammatory burden, which may improve insulin sensitivity and glucose metabolism [115]. Several studies have reported that after periodontal therapy (such as scaling and root planning or more advanced treatments like surgical periodontal therapy), CRP levels, IL-6, and TNF-α levels significantly decrease [116]. This reduction in inflammatory cytokines can help reduce systemic inflammation and improve overall insulin sensitivity [117]. A systematic review and meta-analysis conducted in 2020 investigated the effect of periodontal treatment, specifically scaling and root planning (SRP), in patients with periodontitis and type 2 diabetes (T2D) and demonstrated that SRP significantly impacted both metabolic control and the reduction of systemic inflammation in these patients [118]. A 2022 umbrella review concluded that non-surgical treatment of periodontitis is an efficacious therapy for improving glycemic control in type 2 diabetes mellitus patients, both at 3- and 6-month follow-up [119]. Other studies have shown that periodontal treatment can enhance insulin sensitivity [120]. One study in patients with type 2 diabetes and periodontitis found that after periodontal scaling, patients showed improved insulin sensitivity and better glucose control, reflected in decreased fasting blood glucose levels and improved HbA1c [121]. A 2024 study published in the Journal of Clinical Periodontology demonstrated that periodontal treatment may improve glycemic control in individuals with diabetes, especially in those with hemoglobin A1c levels ≥ 7.0%. [122], suggesting that managing oral health can have broader effects on overall metabolic health.

### 4.2. Molecular and Microbiological Interconnections

Beyond the local effects on the gums, the oral microbiota can influence systemic health through bacteremia and bacteria in the bloodstream [123]. Bacteria from the oral cavity, particularly during episodes of periodontal disease, can enter the bloodstream and travel to distant organs, where they may trigger systemic inflammation [124]. Periodontal bacteria are found in various tissues, such as the heart, liver, and pancreas. This can result in the activation of inflammatory pathways that exacerbate metabolic conditions. Periodontal pathogens in the bloodstream increase C-reactive protein (CRP) levels and other pro-inflammatory cytokines, contributing to chronic low-grade inflammation—a key feature of diabetes and other metabolic disorders [125]. At the molecular level, periodontitis and diabetes share several signaling pathways that mediate inflammation and metabolic dysfunction [126].

1. NF-κB (Nuclear Factor kappa B) pathway

The nuclear factor kappa-light-chain-enhancer of activated B cells (NF-κB) signaling pathway is a pivotal regulator of immune and inflammatory responses [127]. Activated by stimuli such as pro-inflammatory cytokines (e.g., TNF-α, IL-1β), bacterial lipopolysaccharides (LPS), oxidative stress, and advanced glycation end-products (AGEs) [112], NF-κB translocate to the nucleus to promote the expression of genes involved in inflammation and pro-survival [128].

2. Role in Periodontitis and Diabetes mellitus

In periodontitis, NF-κB plays a central role in mediating the chronic inflammatory response to oral pathogens like Porphyromonas gingivalis. LPS from these bacteria activates NF-κB in gingival cells, leading to increased production of pro-inflammatory cytokines such as IL-1β, TNF-α, and IL-6 [129]. This results in the recruitment of immune cells, enhancement of osteoclast activity causing alveolar bone resorption, and impairment of tissue repair due to sustained inflammation [130]. In diabetes, hyperglycemia and elevated AGE levels activate NF-κB, contributing to chronic systemic inflammation. This activation leads to increased production of pro-inflammatory cytokines, which disrupt insulin signaling and promote insulin resistance [131]. Hyperglycemia and other metabolic abnormalities associated with diabetes activate the NF-κB pathway, leading to the overproduction of pro-inflammatory cytokines like TNF-α and IL-6, which interfere with insulin signaling giving rise to mutual amplification of inflammation [132]. The NF-κB pathway contributes to adipocytes and other tissues producing more reactive oxygen species (ROS), which can worsen insulin resistance and glucose intolerance [133]. Additionally, NF-κB activation in endothelial cells contributes to vascular complications, and in pancreatic β-cells, it impairs insulin secretion and promotes apoptosis, exacerbating hyperglycemia [134]. By acting as a central regulator of inflammation in both diseases, NF-κB exemplifies the bidirectional relationship between periodontitis and diabetes. Targeting this pathway therapeutically could mitigate the progression of both conditions.

3. Oxidative Stress and RAGE

Oxidative stress occurs when reactive oxygen species (ROS) overwhelm the body’s antioxidant defenses, leading to cellular damage [135]. The receptor for advanced glycation end products (RAGE) is a multi-ligand receptor that interacts with AGEs and other damage-associated molecular patterns (DAMPs) [136].RAGE activation triggers a cascade of inflammatory responses, including ROS production, and amplifies cytokine release. This creates a feedback loop, enhancing oxidative stress and inflammation, which drives disease progression in conditions such as periodontitis and diabetes [137]. Therefore, it is essential for dental healthcare professionals to be aware of the bidirectional link between diabetes and periodontal diseases. Integrating therapeutic approaches that address both conditions is crucial to improving overall patient health.

## 5. Conclusions and Further Directions

In conclusion, the intricate bidirectional relationship between periodontitis and diabetes underscores the critical need for an integrated approach to managing these chronic diseases. The interplay between these chronic conditions exacerbates systemic inflammation and metabolic dysregulation, significantly reducing patients’ overall well-being and daily functioning. Periodontitis not only exacerbates glycemic dysregulation in diabetes through mechanisms such as inflammation, immune dysfunction, and microbial dysbiosis, but diabetes, in turn, increases susceptibility to and severity of periodontitis. Remember that targeting oral dysbiosis by periodontal therapy, which is not only a readily available treatment but is also cost-effective, can help break the vicious cycle of both these diseases. Simply controlling microbial dysbiosis, the complications that degrade the quality of life, like tooth loss in periodontitis and increased body fat, can be targeted. Here, a multidisciplinary healthcare approach is recommended. When a patient with periodontal disease is identified an effort to make a blood investigation to rule out diabetes should be made by the dentist at the office. Similarly, endocrinologists should advise scaling and root planning treatment to reduce the bacterial load and hence improve glycemic control. In collaboration with the dietician, a patient-specific diet plan with also help tackle both conditions effectively.

Combination therapies that target both conditions will help patients address the complexities of managing diabetes and periodontitis simultaneously. Emerging research on biomarkers like advanced glycation end products (AGEs) and their receptors (RAGEs), as well as therapies targeting pro-inflammatory cytokines, highlights promising avenues for integrated treatment strategies. Healthcare professionals can optimize outcomes for patients affected by both conditions by targeting shared pathways and mitigating chronic inflammation. It is important to consider the social relevance of this topic, given the substantial public health burden posed by the co-occurrence of diabetes and periodontitis, particularly in vulnerable populations. The implementation of integrated clinical practices could not only enhance patient care but also reduce future complications and associated healthcare costs, promoting a more efficient and comprehensive approach.

## Data Availability

All the references are cited in the manuscript; however, we apologize for the omission of any primary citations.

## References

[1] Lenartova M., Tesinska B., Janatova T., Hrebicek O., Mysak J., Janata J., Najmanova L. (2021). The Oral Microbiome in Periodontal Health. Front. Cell. Infect. Microbiol..

[2] Kwon T., Lamster I.B., Levin L. (2021). Current Concepts in the Management of Periodontitis. Int. Dent. J..

[3] Nibali L., Gkranias N., Mainas G., Di Pino A. (2022). Periodontitis and implant complications in diabetes. Periodontol. 2000.

[4] Kumar R., Saha P., Kumar Y., Sahana S., Dubey A., Prakash O. (2020). A review on diabetes mellitus: Type1 & Type2. World J. Pharm. Pharm. Sci..

[5] Ruze R., Liu T., Zou X., Song J., Chen Y., Xu R., Yin X., Xu Q. (2023). Obesity and type 2 diabetes mellitus: Connections in epidemiology, pathogenesis, and treatments. Front. Endocrinol..

[6] Nascimento G.G., Alves-Costa S., Romandini M. (2024). Burden of severe periodontitis and edentulism in 2021, with projections up to 2050: The Global Burden of Disease 2021 study. J. Periodontal Res..

[7] WHO Oral Health. https://www.who.int/news-room/fact-sheets/detail/oral-health.

[8] Fdiworlddental Global Periodontal Health Project. https://www.fdiworlddental.org/gphp.

[9] Ahmad E., Lim S., Lamptey R., Webb D.R., Davies M.J. (2022). Type 2 diabetes. Lancet.

[10] World Health Organization What Are the Consequences of Diabetes?. https://www.emro.who.int/right-teasers/diabetes-info/what-are-the-consequences-of-diabetes.html.

[11] Luong A., Tawfik A.N., Islamoglu H., Gobriel H.S., Ali N., Ansari P., Shah R., Hung T., Patel T., Henson B. (2021). Periodontitis and diabetes mellitus co-morbidity: A molecular dialogue. J. Oral Biosci..

[12] Wang R.P.-H., Huang J., Chan K.W.Y., Leung W.K., Goto T., Ho Y.-S., Chang R.C.-C. (2023). IL-1β and TNF-α play an important role in modulating the risk of periodontitis and Alzheimer’s disease. J. Neuroinflamm..

[13] Rapone B., Ferrara E., Corsalini M., Qorri E., Converti I., Lorusso F., Delvecchio M., Gnoni A., Scacco S., Scarano A. (2021). Inflammatory Status and Glycemic Control Level of Patients with Type 2 Diabetes and Periodontitis: A Randomized Clinical Trial. Int. J. Environ. Res. Public Health.

[14] Yang Y., Sun X., Yang Y., Qie Y. (2024). Insight of the interrelationship and association mechanism between periodontitis and diabetes mellitus. Regen. Ther..

[15] Chawla K., Sawai M.A., Bhardwaj A., Jafri Z., Sultan N. (2024). Prevalence of Periodontitis Based on the American Academy of Periodontology and European Federation of Periodontology 2017 Classification Scheme in Patients Visiting a Tertiary Care Center in New Delhi—An Epidemiological Study. J. Indian Assoc. Public Health Dent..

[16] Abusleme L., Hoare A., Hong B.-Y., Diaz P.I. (2021). Microbial signatures of health, gingivitis, and periodontitis. Periodontol. 2000.

[17] Relvas M., López-Jarana P., Monteiro L., Pacheco J.J., Braga A.C., Salazar F. (2022). Study of Prevalence, Severity and Risk Factors of Periodontal Disease in a Portuguese Population. J. Clin. Med..

[18] La Rosa G.R.M., Chapple I., Polosa R., Pedullà E. (2023). A scoping review of new technologies for dental plaque quantitation: Benefits and limitations. J. Dent..

[19] Di Stefano M., Santonocito S., Polizzi A., Mauceri R., Troiano G., Lo Giudice A., Romano A., Mascitti M., Isola G. (2023). A reciprocal link between oral, gut microbiota during periodontitis: The potential role of probiotics in reducing dysbiosis-induced inflammation. Int. J. Mol. Sci..

[20] Łasica A., Golec P., Laskus A., Zalewska M., Gędaj M., Popowska M. (2024). Periodontitis: Etiology, conventional treatments, and emerging bacteriophage and predatory bacteria therapies. Front. Microbiol..

[21] Buduneli N. (2021). Environmental factors and periodontal microbiome. Periodontol. 2000.

[22] Abdulkareem A.A., Al-Taweel F.B., Al-Sharqi A.J.B., Gul S.S., Sha A., Chapple I.L.C. (2023). Current concepts in the pathogenesis of periodontitis: From symbiosis to dysbiosis. J. Oral Microbiol..

[23] Song B., Zhang Y., Chen L., Zhou T., Huang W., Zhou X., Shao L. (2017). The role of Toll-like receptors in periodontitis. Oral Dis..

[24] Baker J.L., Mark Welch J.L., Kauffman K.M., McLean J.S., He X. (2024). The oral microbiome: Diversity, biogeography and human health. Nat. Rev. Microbiol..

[25] Di Stefano M., Polizzi A., Santonocito S., Romano A., Lombardi T., Isola G. (2022). Impact of Oral Microbiome in Periodontal Health and Periodontitis: A Critical Review on Prevention and Treatment. Int. J. Mol. Sci..

[26] Lee Y.-H., Chung S.W., Auh Q.-S., Hong S.-J., Lee Y.-A., Jung J., Lee G.-J., Park H.J., Shin S.-I., Hong J.-Y. (2021). Progress in Oral Microbiome Related to Oral and Systemic Diseases: An Update. Diagnostics.

[27] Li X., Liu Y., Yang X., Li C., Song Z. (2022). The Oral Microbiota: Community Composition, Influencing Factors, Pathogenesis, and Interventions. Front. Microbiol..

[28] Scannapieco F.A., Dongari-Bagtzoglou A. (2021). Dysbiosis revisited: Understanding the role of the oral microbiome in the pathogenesis of gingivitis and periodontitis: A critical assessment. J. Periodontol..

[29] Fernandes G.V.O., Mosley G.A., Ross W., Dagher A., Martins B.G.d.S., Fernandes J.C.H. (2024). Revisiting Socransky’s complexes: A review suggesting updated new bacterial clusters (GF-MoR Complexes) for periodontal and peri-implant diseases and conditions. Microorganisms.

[30] Qin H., Li G., Xu X., Zhang C., Zhong W., Xu S., Yin Y., Song J. (2022). The role of oral microbiome in periodontitis under diabetes mellitus. J. Oral Microbiol..

[31] Chen Y., Shi T., Li Y., Huang L., Yin D. (2022). Fusobacterium nucleatum: The opportunistic pathogen of periodontal and peri-implant diseases. Front. Microbiol..

[32] Huang Y., Ni S. (2024). Aggregatibacter actinomycetemcomitans with periodontitis and rheumatoid arthritis. Int. Dent. J..

[33] Yakar N., Unlu O., Cen L., Hasturk H., Chen T., Shi W., He X., Kantarci A. (2024). Targeted elimination of Fusobacterium nucleatum alleviates periodontitis. J. Oral Microbiol..

[34] Okamura H., Hirota K., Yoshida K., Weng Y., He Y., Shiotsu N., Ikegame M., Uchida-Fukuhara Y., Tanai A., Guo J. (2021). Outer membrane vesicles of Porphyromonas gingivalis: Novel communication tool and strategy. Jpn. Dent. Sci. Rev..

[35] Gasmi Benahmed A., Kumar Mujawdiya P., Noor S., Gasmi A. (2022). Porphyromonas Gingivalis in the Development of Periodontitis: Impact on Dysbiosis and Inflammation. Arch. Razi Inst..

[36] Moye Z.D., Gormley C.M., Davey M.E. (2019). Galactose Impacts the Size and Intracellular Composition of the Asaccharolytic Oral Pathobiont Porphyromonas gingivalis. Appl. Environ. Microbiol..

[37] Silva I.L., Cascales E. (2021). Molecular strategies underlying Porphyromonas gingivalis virulence. J. Mol. Biol..

[38] Hasegawa Y., Nagano K. (2021). Porphyromonas gingivalis FimA and Mfa1 fimbriae: Current insights on localization, function, biogenesis, and genotype. Jpn. Dent. Sci. Rev..

[39] How K.Y., Song K.P., Chan K.G. (2016). Porphyromonas gingivalis: An overview of periodontopathic pathogen below the gum line. Front. Microbiol..

[40] Marcano R., Rojo M.Á., Cordoba-Diaz D., Garrosa M. (2021). Pathological and therapeutic approach to endotoxin-secreting bacteria involved in periodontal disease. Toxins.

[41] Bostanci N., Belibasakis G.N. (2012). Porphyromonas gingivalis: An invasive and evasive opportunistic oral pathogen. FEMS Microbiol. Lett..

[42] Zhou M., Graves D.T. (2022). Impact of the host response and osteoblast lineage cells on periodontal disease. Front. Immunol..

[43] Cardoso E.M., Reis C., Manzanares-Céspedes M.C. (2018). Chronic periodontitis, inflammatory cytokines, and interrelationship with other chronic diseases. Postgrad. Med..

[44] Groeger S., Meyle J. (2019). Oral Mucosal Epithelial Cells. Front. Immunol..

[45] Akira S., Uematsu S., Takeuchi O. (2006). Pathogen Recognition and Innate Immunity. Cell.

[46] Kawai T., Akira S. (2010). The role of pattern-recognition receptors in innate immunity: Update on Toll-like receptors. Nat. Immunol..

[47] Dias I.H., Marshall L., Lambert P.A., Chapple I.L., Matthews J.B., Griffiths H.R. (2008). Gingipains from Porphyromonas gingivalis increase the chemotactic and respiratory burst-priming properties of the 77-amino-acid interleukin-8 variant. Infect. Immun..

[48] Graves D. (2008). Cytokines That Promote Periodontal Tissue Destruction. J. Periodontol..

[49] Capucetti A., Albano F., Bonecchi R. (2020). Multiple roles for chemokines in neutrophil biology. Front. Immunol..

[50] Lin D., Yang L., Wen L., Lu H., Chen Q., Wang Z. (2021). Crosstalk between the oral microbiota, mucosal immunity, and the epithelial barrier regulates oral mucosal disease pathogenesis. Mucosal Immunol..

[51] Chopra A., Bhat S.G., Sivaraman K. (2020). Porphyromonas gingivalis adopts intricate and unique molecular mechanisms to survive and persist within the host: A critical update. J. Oral Microbiol..

[52] Vitkov L., Muñoz L.E., Schoen J., Knopf J., Schauer C., Minnich B., Herrmann M., Hannig M. (2021). Neutrophils orchestrate the periodontal pocket. Front. Immunol..

[53] Xu X.W., Liu X., Shi C., Sun H.C. (2021). Roles of immune cells and mechanisms of immune responses in periodontitis. Chin. J. Dent. Res..

[54] Moretti J., Blander J.M. (2014). Insights into phagocytosis-coupled activation of pattern recognition receptors and inflammasomes. Curr. Opin. Immunol..

[55] Könönen E., Gursoy M., Gursoy U.K. (2019). Periodontitis: A multifaceted disease of tooth-supporting tissues. J. Clin. Med..

[56] White P., Chicca I., Cooper P., Milward M., Chapple I. (2016). Neutrophil extracellular traps in periodontitis: A web of intrigue. J. Dent. Res..

[57] Birkedal-Hansen H. (1993). Role of matrix metalloproteinases in human periodontal diseases. J. Periodontol..

[58] Mo K., Wang Y., Lu C., Li Z. (2024). Insight into the role of macrophages in periodontitis restoration and development. Virulence.

[59] Yin L., Li X., Hou J. (2022). Macrophages in periodontitis: A dynamic shift between tissue destruction and repair. Jpn. Dent. Sci. Rev..

[60] Luchian I., Goriuc A., Sandu D., Covasa M. (2022). The role of matrix metalloproteinases (MMP-8, MMP-9, MMP-13) in periodontal and peri-implant pathological processes. Int. J. Mol. Sci..

[61] Lambris J.D., Ricklin D., Geisbrecht B.V. (2008). Complement evasion by human pathogens. Nat. Rev. Microbiol..

[62] Hajishengallis G. (2010). Complement and periodontitis. Biochem. Pharmacol..

[63] Damgaard C., Holmstrup P., Van Dyke T.E., Nielsen C.H. (2015). The complement system and its role in the pathogenesis of periodontitis: Current concepts. J. Periodontal Res..

[64] Cheng R., Wu Z., Li M., Shao M., Hu T. (2020). Interleukin-1β is a potential therapeutic target for periodontitis: A narrative review. Int. J. Oral Sci..

[65] Di Benedetto A., Gigante I., Colucci S., Grano M. (2013). Periodontal disease: Linking the primary inflammation to bone loss. J. Immunol. Res..

[66] Chaplin D.D. (2010). Overview of the immune response. J. Allergy Clin. Immunol..

[67] Cardoso E.M., Arosa F.A. (2017). CD8+ T cells in chronic periodontitis: Roles and rules. Front. Immunol..

[68] Bunte K., Beikler T. (2019). Th17 cells and the IL-23/IL-17 axis in the pathogenesis of periodontitis and immune-mediated inflammatory diseases. Int. J. Mol. Sci..

[69] Campbell L., Millhouse E., Malcolm J., Culshaw S. (2016). T cells, teeth and tissue destruction–what do T cells do in periodontal disease?. Mol. Oral Microbiol..

[70] Figueredo C., Lira-Junior R., Love R. (2019). T and B cells in periodontal disease: New functions in a complex scenario. Int. J. Mol. Sci..

[71] Cavalla F., Biguetti C.C., Garlet T.P., Trombone A.P.F., Garlet G.P., Bostanci N., Belibasakis G. (2018). Inflammatory pathways of bone resorption in periodontitis. Pathogenesis of Periodontal Diseases: Biological Concepts for Clinicians.

[72] Sczepanik F.S.C., Grossi M.L., Casati M., Goldberg M., Glogauer M., Fine N., Tenenbaum H.C. (2020). Periodontitis is an inflammatory disease of oxidative stress: We should treat it that way. Periodontol. 2000.

[73] Patil R.T., Dhadse P.V., Salian S.S., Punse S.D. (2024). Role of Oxidative Stress in Periodontal Diseases. Cureus.

[74] Shang J., Liu H., Zheng Y., Zhang Z. (2023). Role of oxidative stress in the relationship between periodontitis and systemic diseases. Front. Physiol..

[75] Katsarou A., Gudbjörnsdottir S., Rawshani A., Dabelea D., Bonifacio E., Anderson B.J., Jacobsen L.M., Schatz D.A., Lernmark Å. (2017). Type 1 diabetes mellitus. Nat. Rev. Dis. Primers.

[76] Marcovecchio M.L. (2017). Complications of acute and chronic hyperglycemia. US Endocrinol..

[77] Deshmukh C.D., Jain A., Nahata B. (2015). Diabetes mellitus: A review. Int. J. Pure Appl. Biosci..

[78] Galicia-Garcia U., Benito-Vicente A., Jebari S., Larrea-Sebal A., Siddiqi H., Uribe K.B., Ostolaza H., Martín C. (2020). Pathophysiology of type 2 diabetes mellitus. Int. J. Mol. Sci..

[79] Chatterjee S., Khunti K., Davies M.J. (2017). Type 2 diabetes. Lancet.

[80] Wilcox G. (2005). Insulin and insulin resistance. Clin. Biochem. Rev..

[81] Kahn B.B., Flier J.S. (2000). Obesity and insulin resistance. J. Clin. Investig..

[82] Chen L., Chen R., Wang H., Liang F. (2015). Mechanisms Linking Inflammation to Insulin Resistance. Int. J. Endocrinol..

[83] Thouvenot K., Turpin T., Taïlé J., Clément K., Meilhac O., Gonthier M.-P. (2022). Links between insulin resistance and periodontal bacteria: Insights on molecular players and therapeutic potential of polyphenols. Biomolecules.

[84] Tilg H., Moschen A.R. (2008). Inflammatory Mechanisms in the Regulation of Insulin Resistance. Mol. Med..

[85] Lawrence M.C. (2021). Understanding insulin and its receptor from their three-dimensional structures. Mol. Metab..

[86] Pei J., Wang B., Wang D. (2022). Current Studies on Molecular Mechanisms of Insulin Resistance. J. Diabetes Res..

[87] Borst S.E. (2004). The role of TNF-α in insulin resistance. Endocrine.

[88] Hotamisligil G. (1999). Mechanisms of TNF-α-induced insulin resistance. Exp. Clin. Endocrinol. Diabetes.

[89] Yaribeygi H., Farrokhi F.R., Butler A.E., Sahebkar A. (2019). Insulin resistance: Review of the underlying molecular mechanisms. J. Cell. Physiol..

[90] Bao S., Wang X., Cho S.B., Wu Y.-L., Wei C., Han S., Bao L., Wu Q., Ao W., Nan J.-X. (2021). Agriophyllum oligosaccharides ameliorate diabetic insulin resistance through INS-R/IRS/Glut4-mediated insulin pathway in db/db mice and MIN6 cells. Front. Pharmacol..

[91] Goh S.-Y., Cooper M.E. (2008). The role of advanced glycation end products in progression and complications of diabetes. J. Clin. Endocrinol. Metab..

[92] Kolb H., Mandrup-Poulsen T. (2010). The global diabetes epidemic as a consequence of lifestyle-induced low-grade inflammation. Diabetologia.

[93] Touch S., Clément K., André S. (2017). T cell populations and functions are altered in human obesity and type 2 diabetes. Curr. Diabetes Rep..

[94] Shi N., Kong C., Yuan L., Liu L., Zhao K., Lv J., Wang X. (2023). The bidirectional relationship between periodontitis and diabetes: New prospects for stem cell-derived exosomes. Biomed. Pharmacother..

[95] Mata A.D., Marques D., Rocha S., Francisco H., Santos C., Mesquita M.F., Singh J. (2004). Effects of diabetes mellitus on salivary secretion and its composition in the human. Mol. Cell. Biochem..

[96] Jafar N., Edriss H., Nugent K. (2016). The effect of short-term hyperglycemia on the innate immune system. Am. J. Med. Sci..

[97] van Niekerk G., Davis T., Patterton H.G., Engelbrecht A.M. (2019). How Does Inflammation-Induced Hyperglycemia Cause Mitochondrial Dysfunction in Immune Cells?. Bioessays.

[98] Gonzalez A.C.d.O., Costa T.F., Andrade Z.d.A., Medrado A.R.A.P. (2016). Wound healing—A literature review. An. Bras. Dermatol..

[99] Wang G., Yang F., Zhou W., Xiao N., Luo M., Tang Z. (2023). The initiation of oxidative stress and therapeutic strategies in wound healing. Biomed. Pharmacother..

[100] Plemmenos G., Evangeliou E., Polizogopoulos N., Chalazias A., Deligianni M., Piperi C. (2021). Central regulatory role of cytokines in periodontitis and targeting options. Curr. Med. Chem..

[101] Kırkgöz T., Acar S., Küme T., Hilal Kırkgöz H., Tabanlı G., Nalbantoğlu Ö., Yılmaz Ü., Ünalp A., Özkan B. (2024). Evaluation of Serum Advanced Glycation End Product Levels and Microvascular Complications in Children and Adolescents with Type 1 Diabetes Mellitus. Turk. Arch. Pediatr..

[102] Khalid M., Petroianu G., Adem A. (2022). Advanced glycation end products and diabetes mellitus: Mechanisms and perspectives. Biomolecules.

[103] Chopra A., Jayasinghe T.N., Eberhard J. (2022). Are inflamed periodontal tissues endogenous source of advanced glycation end-products (AGEs) in individuals with and without diabetes mellitus? A systematic review. Biomolecules.

[104] Dhande S., Khan M., Muglikar S., Chaudhari S., Jangale S.A., Jangale A.G. (2022). Diabetes and Periodontal Disease: The Reciprocal Relationship. J. Gen. Dent..

[105] Mirnic J., Djuric M., Brkic S., Gusic I., Stojilkovic M., Tadic A., Veljovic T. (2024). Pathogenic mechanisms that may link periodontal disease and type 2 diabetes mellitus—the role of oxidative stress. Int. J. Mol. Sci..

[106] Ellingson A., Pancheri N., Schiele N. (2022). Regulators of collagen crosslinking in developing and adult tendons. Eur. Cells Mater..

[107] Klonoff D.C., Aaron R.E., Tian T., DuNova A.Y., Pandey A., Rhee C., Fleming G.A., Sacks D.B., Pop-Busui R., Kerr D. (2024). Advanced Glycation Endproducts: A Marker of Long-term Exposure to Glycemia. J. Diabetes Sci. Technol..

[108] Ilea A., Băbţan A.M., Boşca B.A., Crişan M., Petrescu N.B., Collino M., Sainz R.M., Gerlach J.Q., Câmpian R.S. (2018). Advanced glycation end products (AGEs) in oral pathology. Arch. Oral Biol..

[109] Hajishengallis G., Chavakis T. (2021). Local and systemic mechanisms linking periodontal disease and inflammatory comorbidities. Nat. Rev. Immunol..

[110] Genco R.J., Graziani F., Hasturk H. (2020). Effects of periodontal disease on glycemic control, complications, and incidence of diabetes mellitus. Periodontol. 2000.

[111] Preshaw P.M., Bissett S.M. (2019). Periodontitis and diabetes. Br. Dent. J..

[112] Chen Z., Lang G., Xu X., Liang X., Han Y., Han Y. (2024). The role of NF-kappaB in the inflammatory processes related to dental caries, pulpitis, apical periodontitis, and periodontitis—A narrative review. PeerJ.

[113] Bains V.K., Mahendra J., Mahendra L., Mittal M., Valli G. (2022). Markers, Pathways, and Current Evidence for Periodontitis-associated Insulin Resistance: A Narrative Review. J. Int. Soc. Prev. Community Dent..

[114] Inoue M., Sakanaka A., Katakami N., Furuno M., Nishizawa H., Omori K., Taya N., Ishikawa A., Mayumi S., Tanaka Isomura E. (2024). Periodontal tissue susceptibility to glycaemic control in type 2 diabetes. Diabetes Obes. Metab..

[115] Hasturk H., Kantarci A. (2015). Activation and resolution of periodontal inflammation and its systemic impact. Periodontol. 2000.

[116] Eivazi M., Falahi N., Eivazi N., Eivazi M.A., Raygani A.V., Rezaei F. (2017). The Effect of Scaling and Root Planning on Salivary TNF-α and IL-1α Concentrations in Patients with Chronic Periodontitis. Open Dent. J..

[117] Schenk S., Saberi M., Olefsky J.M. (2008). Insulin sensitivity: Modulation by nutrients and inflammation. J. Clin. Investig..

[118] Baeza M., Morales A., Cisterna C., Cavalla F., Jara G., Isamitt Y., Pino P., Gamonal J. (2020). Effect of periodontal treatment in patients with periodontitis and diabetes: Systematic review and meta-analysis. J. Appl. Oral Sci..

[119] Di Domenico G.L., Minoli M., Discepoli N., Ambrosi A., de Sanctis M. (2023). Effectiveness of periodontal treatment to improve glycemic control: An umbrella review. Acta Diabetol..

[120] Sgolastra F., Severino M., Pietropaoli D., Gatto R., Monaco A. (2013). Effectiveness of Periodontal Treatment to Improve Metabolic Control in Patients With Chronic Periodontitis and Type 2 Diabetes: A Meta-Analysis of Randomized Clinical Trials. J. Periodontol..

[121] Teeuw W.J., Gerdes V.E.A., Loos B.G. (2010). Effect of Periodontal Treatment on Glycemic Control of Diabetic Patients: A systematic review and meta-analysis. Diabetes Care.

[122] Sato M., Ono S., Yamana H., Okada A., Ishimaru M., Ono Y., Iwasaki M., Aida J., Yasunaga H. (2024). Effect of periodontal therapy on glycaemic control in type 2 diabetes. J. Clin. Periodontol..

[123] Sedghi L., DiMassa V., Harrington A., Lynch S.V., Kapila Y.L. (2021). The oral microbiome: Role of key organisms and complex networks in oral health and disease. Periodontol. 2000.

[124] Kitamoto S., Nagao-Kitamoto H., Hein R., Schmidt T.M., Kamada N. (2020). The Bacterial Connection between the Oral Cavity and the Gut Diseases. J. Dent. Res..

[125] Cecoro G., Annunziata M., Iuorio M.T., Nastri L., Guida L. (2020). Periodontitis, Low-Grade Inflammation and Systemic Health: A Scoping Review. Medicina.

[126] Portes J., Bullón B., Quiles J.L., Battino M., Bullón P. (2021). Diabetes Mellitus and Periodontitis Share Intracellular Disorders as the Main Meeting Point. Cells.

[127] Liu T., Zhang L., Joo D., Sun S.-C. (2017). NF-κB signaling in inflammation. Signal Transduct. Target. Ther..

[128] Liu D., Zhong Z., Karin M. (2022). NF-κB: A double-edged sword controlling inflammation. Biomedicines.

[129] Charoensaensuk V., Chen Y.-C., Lin Y.-H., Ou K.-L., Yang L.-Y., Lu D.-Y. (2021). Porphyromonas gingivalis induces proinflammatory cytokine expression leading to apoptotic death through the oxidative stress/NF-κB pathway in brain endothelial cells. Cells.

[130] Supandi S., Elvandari A., Bargowo L., Wijaksana I. (2023). Nigella sativa extract on gingival epithelium exposed to LPS Porphyromonas gingivalis and its impact on the expression of TLR-4 and NF-kB in vivo study. Nat. Life Sci. Commun..

[131] Zand H., Morshedzadeh N., Naghashian F. (2017). Signaling pathways linking inflammation to insulin resistance. Diabetes Metab. Syndr. Clin. Res. Rev..

[132] Suryavanshi S.V., Kulkarni Y.A. (2017). NF-κβ: A Potential Target in the Management of Vascular Complications of Diabetes. Front. Pharmacol..

[133] Zatterale F., Longo M., Naderi J., Raciti G.A., Desiderio A., Miele C., Beguinot F. (2020). Chronic Adipose Tissue Inflammation Linking Obesity to Insulin Resistance and Type 2 Diabetes. Front. Physiol..

[134] Krinock M.J., Singhal N.S. (2021). Diabetes, stroke, and neuroresilience: Looking beyond hyperglycemia. Ann. N. Y. Acad. Sci..

[135] Jaganjac M., Milkovic L., Zarkovic N., Zarkovic K. (2022). Oxidative stress and regeneration. Free. Radic. Biol. Med..

[136] Zhu X., Huang H., Zhao L. (2022). PAMPs and DAMPs as the bridge between periodontitis and atherosclerosis: The potential therapeutic targets. Front. Cell Dev. Biol..

[137] Yamamoto Y., Yamamoto H. (2013). RAGE-mediated inflammation, type 2 diabetes, and diabetic vascular complication. Front. Endocrinol..

